# An Enviable Principality

**Published:** 1899-11

**Authors:** 


### An Enviable Principality.

We are not accustomed to think of an Indian prince, ruling some tributary state in British India, as a man of advanced ideas. In the *Indian Medical Record*, however, we read that the Thakore Sahib of Gondal, the ruler of a principality of some thousand square miles, is a medical graduate of Edinburgh University and the author of a medical work; that in his dominions sanitary regulations are thoroughly carried out, vaccination enforced, hospitals and dispensaries maintained, and an efficient health organization, that enforces local sanitation and reports reliable statistics exists. Inquests are made over every accidental, violent or suspicious case of death, food and water infection, etc., in short, everything apparently that we expect to find in all these particulars in a highly civilized Caucasian community. The only Oriental feature in the report is the order to remove a village bodily from an unhealthy locality to a better one, which was carried out and "hailed with delight by the villagers concerned," who received "every facility, pecuniary or otherwise," in the removal. Notwithstanding the fact that the principality appears to be within the plague-stricken section of India, it has escaped the pest, owing to the extreme precautions taken. It has an increasing birth-rate, with a decreasing death-rate to top off its sanitary prosperity. Agriculture is aided by the government, which maintains public gardens and an experimental farm, and there is an "agricultural association" under its direct patronage. The exports are increasing and the imports decreasing, while the gross public revenue exceeds expenditures nearly 50 percent. If all the minor states in British India were like the one here described, that section of the world would be a most enviable one in many, if not all, respects.—*The Journal.*

